# The novel diagnostic value and characteristic imaging features of CT dacryocystography in primary lacrimal canaliculitis

**DOI:** 10.1038/s41598-025-24561-z

**Published:** 2025-11-07

**Authors:** Lihong Yang, Zhi Yin, Hua Sun, Hongxun Li, Peng Wang, Yuchuan Wang, Lei Zhang, Jiagen Li, Yun Zhao, Ye Pan, Hong Zhao, Zhenbiao Xu, Jing Jing

**Affiliations:** 1 Tianjin Eye Hospital, Tianjin Key Laboratory of Ophthalmology and Vision Science, Affiliated Eye Hospital of NanKai University, Clinical College of Ophthalmology of Tianjin Medical University, Tianjin, 300020 China; 2 Beijing Tongren Hospital, Beijing Institute of Ophthalmology, Beijing Tongren Eye Center, Capital Medical University, Beijing, 100005 China; 3 Huanxian People’s Hospital, Qingyang City, 745700 Gansu Province China

**Keywords:** Primary lacrimal canaliculitis, Computed tomographic dacryocystography (CT-DCG), Imaging features, Dacryoendoscopy, Diagnostic value, Diseases, Medical research

## Abstract

To analyze the imaging characteristics and evaluate the diagnostic value of computed tomographic dacryocystography (CT-DCG) in primary canaliculitis. This is a retrospective study from three medical centers. We enrolled 126 primary canaliculitis patients (126 eyes) and 108 non-canaliculitis subjects (134 eyes). All patients with canaliculitis underwent CT-DCG, dacryoendoscopy examination, microbiologic culture, histopathologic staining and canaliculotomy to confirm the definitive diagnosis. Non-canaliculitis cases were diagnosed via dacryoendoscopy and CT-DCG. Demographics, clinical features, and CT-DCG imaging features of primary canaliculitis were evaluated. The diagnostic performance, including sensitivity, specificity, and inter-observer agreement, was also assessed. In the canaliculitis group, the main imaging characteristics of CT-DCG included filling defects, dilation, raggedness, beading, and diverticula formation. Filling defects can serve as a distinctive diagnostic feature of canaliculitis. In our series, CT-DCG showed excellent diagnostic performance with a sensitivity of 94.4%, specificity of 98.5%, and high inter-observer agreement (Cohen’s kappa = 0.85). CT-DCG demonstrates distinct imaging features that enable accurate diagnosis of primary lacrimal canaliculitis. It overcomes limitations of traditional methods, provides comprehensive LDS visualization, and offers valuable preoperative information, establishing it as a superior diagnostic tool.

## Introduction

Primary lacrimal canaliculitis is a chronic infection of the proximal lacrimal pathway, accounting for approximately 2% of all lacrimal system diseases^1,2^. Its typical symptoms include a pouting punctum, punctal discharge, conjunctival congestion, medial canthal erythema, and epiphora. It often presents with clinical manifestations overlapping with other ocular conditions—including chronic conjunctivitis, dacryocystitis, blepharitis, and chalazion^3,4^. This overlap, coupled with its relative rarity and occasional atypical presentation, leads to high misdiagnosis rates (45%–100% in previous reports)^5^, resulting in prolonged disease course, inappropriate treatments, and potential complications. Therefore, accurate and timely diagnosis is critical for improving patient outcomes, particularly in cases with ambiguous symptoms where diagnosis remains elusive.

Existing diagnostic modalities for lacrimal canaliculitis include microbiological culture, histopathological examination, ultrasound biomicroscopy (UBM), dacryoendoscopy, and traditional dacryocystography^6–10^. However, these methods have notable limitations: microbiological culture has low sensitivity; Histopathological examination is limited by its complex procedures and low detection rates for specific species (e.g., Actinomyces); UBM and dacryoendoscopy are restricted in visualizing the entire lacrimal drainage system (LDS) or are operator-dependent; and conventional imaging techniques (e.g., X-ray dacryocystography) lack sufficient resolution to detect subtle abnormalities^5,11–13^.

Computed tomographic dacryocystography (CT-DCG) has emerged as a non-invasive, high-resolution imaging tool for evaluating the LDS, offering clear visualization of structural details^14^. A preliminary study documented some characteristics of CT-DCG images in 4 cases of secondary canaliculitis^15^. However, systematic investigations into CT-DCG findings in primary canaliculitis remain absent.

To address this gap, we conducted a multi-center retrospective study to analyze the distinct CT-DCG imaging features of primary lacrimal canaliculitis and systematically evaluate its diagnostic value. By comparing CT-DCG findings with gold-standard diagnoses (confirmed via dacryoendoscopy, surgery, microbiology, and/or histopathology), we aimed to establish its role in overcoming the limitations of traditional methods and improving diagnostic accuracy for this frequently misdiagnosed condition.

## Materials and methods

### Patients

This retrospective study was approved by the Ethics Committee of Tianjin Eye Hospital and conducted in accordance with the Declaration of Helsinki for research involving human subjects. All subjects or their legal guardians provided written informed consent to participate.

Patients were recruited from Tianjin Eye Hospital, Beijing Tongren Hospital, and Huanxian People’s Hospital of Gansu Province, with data collected between August 2021 and July 2024. The inclusion criteria were: presence of punctal discharge or concretions (spontaneous, upon massage, or upon irrigation); and other clinical manifestations suggestive of canaliculitis including pouting punctum, recurrent conjunctivitis, medial canthal erythema, and unexplained epiphora. The exclusion criteria were: tumors in medial canthal region, previous lacrimal surgeries; and secondary canaliculitis (e.g., punctal or intracanalicular plug-related canaliculitis).

This study included consecutive medical records of primary canaliculitis patients who had undergone CT-DCG, dacryoendoscopy, microbiological culture, histopathological staining, and canaliculotomy for definitive diagnosis confirmation. It also includes the consecutive medical records of non-canaliculitis patients—who could not be ruled out for canaliculitis based on the initial slit-lamp examination and lacrimal irrigation, but were finally excluded from the diagnosis of canaliculitis after evaluation via dacryoendoscopy and CT-DCG.

### CT-DCG acquisition

0.5-1.0mL of water-soluble contrast medium (Iohexol, 755 mg/mL) was slowly injected into the examined LDS under low pressure via both the inferior and superior puncta. Prior to the injection, topical anesthetic drops (e.g., 0.5% proparacaine hydrochloride) were administered to the ocular surface and lacrimal puncta. Injection was stopped when slight reflux of the contrast medium occurred or the patient reported mild discomfort, with care taken to avoid excessive pressure on the lacrimal system. Orbital plain CT scanning was performed immediately after contrast medium injection using a Lightspeed VCT 64-slice spiral CT (GE), Somatom Definition AS 64-slice spiral CT (Siemens), or Philips IQon Spectral CT. No iatrogenic dacryocystitis or other contrast medium-related complications were observed in this cohort.

The scanning baseline was the auriculoorbital line, perpendicular to the CT machine bed, with the scanning range extending from the frontal sinus to the nasal floor. Scanning parameters included a slice thickness of 0.6 mm, reconstruction thickness of 0.6 mm, voltage of 120 kV, and tube current of 150–220 mA. Axial image data were reconstructed into coronal and parasagittal images along the axis of the canaliculus to ensure clear visualization. Reconstruction was performed using a high-resolution algorithm (e.g., bone or soft-tissue algorithm) to optimize visualization of the lacrimal canaliculi and surrounding soft tissues. CT-DCG images were processed using a GE Advantage Windows workstation with a window width of 2000 HU and a window level of 500 HU.

### Diagnosis and data collection

The diagnosis of canaliculitis was initially presumed based on patients’ clinical histories and findings, with subsequent confirmation by surgical findings, dacryoendoscopy, microbiological analysis, and/or histopathological diagnosis—considered the gold standard.

The following data were collected: demographic information, clinical symptoms and manifestations, slit-lamp photographs, previous diagnoses, duration of misdiagnosis, lacrimal irrigation results, CT-DCG imaging characteristics, intraoperative findings, dacryoendoscopic images, and microbiological and histological analyses.

Two experienced radiologists independently evaluated all CT-DCG imaging features using the workstation. When there were inconsistencies in the description of imaging features or diagnoses, a final consensus was reached through consultation or by seeking guidance from the chief radiologist. Notably, they remained blind to both the clinical information related to the analyzed images and whether the corresponding cases belonged to the canaliculitis group or the non-canaliculitis group, thereby ensuring an unbiased assessment.

Lacrimal syringing was used as the gold standard for evaluating LDS patency. Briefly, fluid regurgitation ≤ 20% was classified as freely patent; >20% as LDS stenosis; and complete regurgitation as LDS obstruction^16^.

### Statistical analyses

Statistical analyses were performed using SPSS 26.0 software. Continuous variables were described as the mean ± standard deviation (SD) or median (range), and comparisons between groups were conducted using the two-tailed Student’s t-test. Categorical variables were presented as frequencies (n (%)) and compared using the two-tailed chi-square test or Fisher’s exact test, as appropriate.

Interobserver reliability for categorical imaging features (e.g., presence/absence of canaliculitis, filling defects, dilation, raggedness, beading, and diverticula formation) was evaluated using Cohen’s kappa coefficient. A kappa value > 0.60 was considered indicative of substantial agreement. Agreement between each diagnostic method (CT-DCG, dacryoendoscopy, and Slit-lamp) and the gold standard was assessed using Cohen’s kappa test. Diagnostic performance metrics (sensitivity, specificity, positive predictive value [PPV], negative predictive value [NPV], and accuracy) were calculated using 2 × 2 contingency tables. All statistical tests were two-sided, with a p-value < 0.05 considered statistically significant.

## Results

### Demographic data and clinical characteristics

A total of 234 subjects (involving 260 eyes) were enrolled in this study, including 126 subjects (126 eyes) of primary canaliculitis and 108 subjects (134 eyes) of non-canaliculitis. The demographic data and clinical characteristics are summarized in Table 1.

**Table 1:** Demographic data and clinical characteristics of the primary canaliculitis group and the non-canaliculitis group.

	Canaliculitis (126 subjects, 126 eyes)	Non-canaliculitis (108 subjects,134 eyes)	*P* value
Gender			0.058^b^
Male	22 (17.5%)	30 (27.8%)	
Female	104 82.5%)	78 (72.2%)	
Age (years, range)	63.1 ± 10.8 (9–88)	59.3 ± 8.4 (43–76)	0.056 ^a^
Laterality			0.081^b^
Right	55 (43.7%)	73 (54.5%)	
Left	71 (56.3%)	61 (45.5%)	
Location			**< 0.001** ^**b**^
Upper only	24 (19.0%)	33 (24.6%)	
Lower only	91 (72.2%)	59 (44.0%)	
Both	11 (8.7%)	42 (31.3%)	
Mean time lapse to correct diagnosis (months, range)	16.2 ± 10.5 (1-120)	2.6 ± 2.1 (0.5–10)	**0.000** ^**a**^

^a^Two-tailed t-test. ^b^Chi-square test. Values with *P* < 0.05 are shown in bold.

**Fig. 1:**
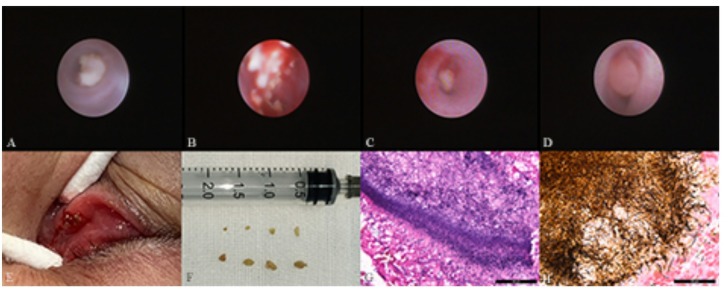
Dacryoendoscopic examination, intraoperative and histological findings of primary canaliculitis. (A) Dacryoendoscopy showing concretions in the central lumen of canaliculi and canalicular dilation. (B) Fragmental concretions in close proximity to the canalicular lumen wall. (C) Concretions at the distal end of the canaliculus close to the common canaliculus. (D) Granulation tissue in the canaliculus lumen. (E) Concretions scraped from the dilated vertical segment of the canaliculus. (F) Concretions detected during canaliculotomy. (G) Giemsa staining of concretions showing blue hyphae and colonies (magnification ×400). (H) Gomori methenamine silver stain showing black hyphae and colonies (magnification × 400). Histological examination revealing typical features of Actinomyces infection. The features are described as interwoven hyphae at the center and radially arranged club-shaped long hyphae at the periphery.

In the canaliculitis group, all patients presented with unilateral eye involvement. The lower canaliculus was more frequently affected (91, 72.2%). Eleven patients (8.7%) had involvement of both the superior and inferior canaliculi. Four patients exhibited cutaneous fistulae in the lacrimal sac region, with visible secretions overflowing from the fistulae. Discharge, concretions, and granulation tissues expressed from the canaliculi or collected during surgery were sent for microbiological evaluation and histopathologic staining. Microbiological culture results were positive in 53 canaliculi from 53 eyes (42.1%) out of 126 eyes, including: Streptococcus species in 23 eyes (18.3%), Actinomyces species in 15 eyes (11.9%), Staphylococcus species in 12 eyes (9.5%), as well as nonspecific Gram-positive organisms, Proteus mirabilis, and Bacillus species occurring in 1 eye each. Histopathological examination of concretions using Gomori methenamine silver staining and Giemsa staining revealed Actinomyces species in 20 canaliculi from 16 eyes (Fig. 1G and H). The remaining cases were definitively diagnosed by detecting concretions or granulation tissues during surgery or dacryoendoscopy.

**Fig. 2:**
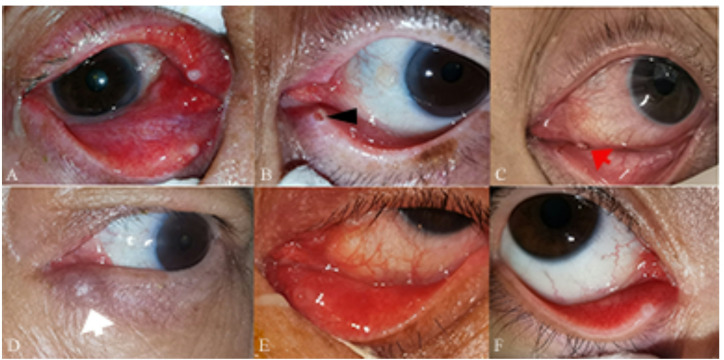
Clinical manifestations of the primary canaliculitis. (A)The upper and lower canaliculitis in the right eye, showing conjunctival congestion, punctal edema, mucopurulent discharge. (B) A granuloma mass protruding from the lower punctum (black arrow). (C) The punctal regurgitation of concretions under pressure application (red arrow). (D)The palpable thickened canaliculus (white arrow). (E)A swollen and pouting punctum accompanied by conjunctival congestion. (F)The atypical clinical feature of primary canaliculitis.

The main complaints and clinical manifestations of the canaliculitis group are summarized in Table 2. Representative clinical findings, such as punctal pouting and conjunctival congestion, are illustrated in Fig. 2. Concretions were not observed in 4 canaliculi: 3 of these contained only granulation tissues, while 1 showed no specific findings. Notably, the presence of concretions was specific for diagnosing canaliculitis compared with the non-canaliculitis group.

**Table 2:** Clinical presentations of the primary canaliculitis group and the non-canaliculitis group.

	Canaliculitis (126 subjects, 126 eyes)	Non-canaliculitis (108 subjects, 134 eyes)	*P* value
Symptoms			
Epiphora	87 (69.0%)	84 (62.7%)	0.280^a^
Mucopurulent discharge	103 (81.7%)	69 (51.5%)	**< 0.001** ^**a**^
Blood-tinged discharge	8 (6.3%)	0	**0.003** ^**b**^
Pain	43 (34.1%)	4 (3.0%)	**< 0.001** ^**a**^
Irritation	85 (67.5%)	53 (39.6%)	**< 0.001** ^**a**^
Clinical signs			
Medial Canthal Erythema	46 (36.5%)	7 (5.2%)	**< 0.001** ^**a**^
Granuloma mass protruding from the punctum	13 (10.3%)	0	**< 0.001** ^**b**^
Conjunctival Congestion	72 (57.1%)	82 (61.2%)	0.506 ^a^
Pouting punctum	56 (44.4%)	5 (3.7%)	**< 0.001** ^**a**^
Punctal regurgitation of concretions under expression	62 (49.2%)	0	**< 0.001** ^**b**^
Palpable thickened canaliculus	49 (38.9%)	0	**< 0.001** ^**b**^
Syringing			**< 0.001** ^**a**^
Patent	106 (84.1%)	73 (54.5%)	
Stenosis	8 (6.3%)	22 (16.4%)	
LDS obstructed	12 (9.5%)	39 (29.1%)	

^a^Chi-square test. ^b^Fisher’s exact test. Values with *P* < 0.05 are shown in bold.

LDS(lacrimal drainage system).

In the non-canaliculitis group, the final diagnoses included: conjunctivitis (38 eyes, 28.4%), blepharitis (32 eyes, 23.9%), dacryocystitis (21 eyes, 15.7%), canalicular cysts (18 eyes, 13.4%), chalazia (14 eyes, 10.4%), and hordeola (11 eyes, 8.2%).

### CT-DCG imaging features of primary canaliculitis

To ensure the reliability of imaging feature analysis, interobserver agreement for feature identification was evaluated. The overall Cohen’s kappa coefficient for all imaging features was 0.85, indicating excellent consistency. For features with relatively lower agreement (kappa < 0.6), consensus was reached through consultation or guidance from the chief radiologist, further minimizing potential errors. These measures confirmed the robustness of the extracted imaging features and supported the validity of subsequent analyses.

In the canaliculitis group, 126 patients (involving 137 canaliculi) presented with unilateral involvement. The imaging features of primary canaliculitis on CT-DCG are summarized in Table 3. CT-DCG successfully identified 130 abnormal canaliculi in the canaliculitis group and 132 eyes in the non-canaliculitis group, yielding a sensitivity of 94.4% and a specificity of 98.5% for diagnosing primary canaliculitis (Table 4).

**Table 3:** CT-DCG imaging features of primary canaliculitis (126 patients, 137 canaliculi).

CT-DCG imaging features		No. (%)	
dilatation of the canaliculus		127 (92.7%)	
Filling defects	Only filling defects close to canalicular lumen wall	110 (80.3%)	38 (27.7%)
	Only central radiolucency with surrounding enhancement		47 (34.3%)
	Both		25 (18.2%)
Beading appearance		41 (29.9%)	
Small diverticula		13 (9.5%)	
Raggedness		35(25.5%)	

**Table 4:** Diagnostic performance of three examinations in primary lacrimal canaliculitis and non-canaliculitis.

Examinations	Canaliculitis (126 subjects, 126 eyes)					Non-canaliculitis (108 subjects, 134 eyes)		Reliability		Validity	
	Correct diagnosis	Misdiagnosis in other hospital				Correct diagnosis	Misdiagnosis as canaliculitis	Kappa Value^a^	Consistency^b^	Sensitivity (95%CI)	Specificity (95%CI)
	Canaliculitis 35 (27.8%)	Dacryocystitis 24 (19.0%)	Conjunctivitis 45 (35.7%)	Chalazion 13(10.3%)	Blepharitis 9 (7.1%)						
Confirmed by Slit-lamp	35	15	24	10	5	95	39	0.42	Mediocre	70.6%	70.9%
Confirmed by Dacryoendoscopy	35	20	39	10	7	134	0	0.88	Good	88.1%	100%
Confirmed by CT-DCG	35	24	40	13	7	132	2	0.93	Very Good	94.4%	98.5%

^a^Kappa value: calculated by Cohen’s kappa test. ^b^Consistency rating: very good (kappa ≥ 0.9), good (0.75 ≤ kappa < 0.9), mediocre (0.4 ≤ kappa < 0.75), poor (kappa < 0.4). 95% CI = 95% confidence interval.

**Fig. 3:**
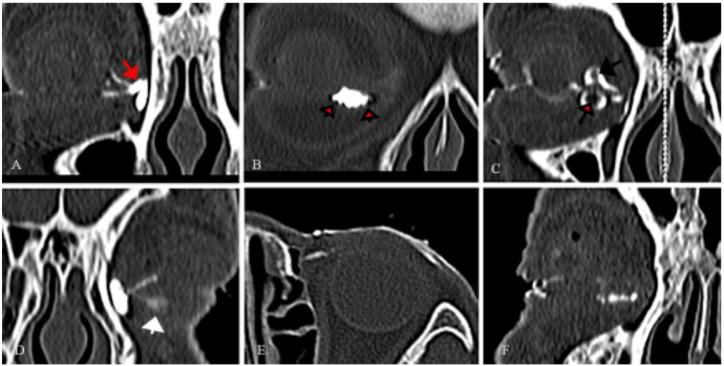
CT-DCG imaging features of primary canaliculitis. (A) The dilation of the common canaliculus (red arrow). (B) Canalicular dilation towards the temporal side with filling defects in close proximity to the canalicular lumen wall (short black arrow). (C) Both the upper and lower canaliculi showed canalicular dilation and central radiolucency with marked surrounding enhancement (long black arrow). (D) Temporal dilation of the lower canaliculus, accompanied by small filling defects (white arrow). (E) Raggedness of the canalicular lumen wall. (F) A beading appearance of the lower canaliculus.

**Fig. 4:**
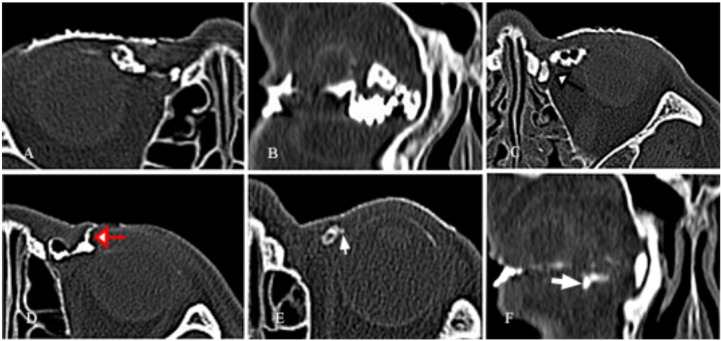
CT-DCG imaging features of primary canaliculitis.(A) Canalicular dilation towards the temporal side with both types of filling defects. (B) Dilations involved the entire length of the upper and lower canaliculi with both types of filling defects. (C) Dilation and filling defects of the canaliculus accompanied by dilation of the lacrimal sac (black arrow). (D) A skin fistula connected to the affected inferior canaliculus (red arrow). (E) The diverticulum formed on the temporal side of the canaliculus (thin white arrow). (F) The diverticulum formed below the vertical segment of the lower canaliculus (thick white arrow).

Within the canaliculitis group, 127 canaliculi exhibited dilation. Among these dilated canaliculi: 56 showed dilation near the vertical segment, extending to varying degrees toward the temporal side (Fig. 3D); 40 demonstrated dilation in the proximal two-thirds of the horizontal segment (Fig. 3F); 10 had dilation at the distal end, adjacent to the common canaliculus (Fig. 3A); and 21 displayed dilation involving the entire length of the canaliculus (Fig. 4B).

Filling defects were observed in 110 canaliculi, categorized as follows: 38 defects adjacent to the canalicular lumen wall (Fig. 3B); 47 with central radiolucency and marked surrounding enhancement (Fig. 3C); and 25 with both types of defects (Fig. 4A and B).

Additional imaging findings included: a “beading” appearance in 41 canaliculi (Fig. 3F); small diverticula in 13 canaliculi (Fig. 4E and F); a “ragged” lumen wall in 35 canaliculi (Fig. 3E).

CT-DCG detected dilation, filling defects, beading, or small diverticula in 127 of the 137 affected canaliculi; only 3 canaliculi showed isolated lumen wall raggedness, which was suggestive of the diagnosis.

Subsequent dacryoendoscopic examination revealed that the 110 filling defects in affected canaliculi corresponded to 108 concretions and 2 granulation tissues (Fig. 1A-D). All cases with filling defects were accompanied by varying degrees of lacrimal canalicular dilation. Meanwhile, canaliculi with beading changes were found to contain multiple focal lesions, predominantly concretions.

One case showed distal obstruction of the affected canaliculus. Eleven patients suspected of canaliculitis exhibited canalicular dilation and filling defects on imaging, along with lacrimal sac dilation and nasolacrimal duct obstruction (Fig. 4C). Combined with clinical manifestations, these 11 cases were confirmed as dacryocystitis complicated by canaliculitis.

### Misdiagnosis

Among the 126 patients (126 eyes) with canaliculitis, 91 cases (91 eyes) had been previously misdiagnosed as dacryocystitis, conjunctivitis, chalazion, or blepharitis in other hospitals. The mean duration from symptom onset to definitive diagnosis was 16.2 months (median: 10 months; range: 1–120 months).

An analysis of diagnostic performance across different examination methods revealed the following:

Slit-lamp examination showed mediocre consistency with the gold standard (kappa = 0.42), with a sensitivity of 70.6% and specificity of 70.9%. It missed 37 cases of canaliculitis and incorrectly diagnosed 39 cases as canaliculitis, yielding a positive predictive value (PPV) of 69.53% and a negative predictive value (NPV) of 71.97%.

Notably, among the 11 cases of combined superior and inferior canaliculitis in this series, slit-lamp examination identified 2 cases with involvement of both canaliculi. For dacryoendoscopy, 3 of these 11 cases underwent concurrent examination of both the superior and inferior canaliculi, which successfully confirmed combined involvement; the remaining 8 cases did not undergo examination of the contralateral canaliculus due to atypical symptoms, leading to missed diagnoses of combined disease. In contrast, CT-DCG accurately detected all 11 cases of combined superior and inferior canaliculitis.

Dacryoendoscopy demonstrated good consistency with the gold standard (kappa = 0.88), with a sensitivity of 88.1% and specificity of 100%, but still missed 15 cases of canaliculitis. Among these missed cases, 12 were attributed to difficulties in visualizing concretions and granulation tissues in the vertical segment of the canaliculus. Due to its proximity to the lacrimal punctum, this segment constitutes a relative blind spot for dacryoendoscopy. The remaining 3 cases involved small concretions, where temporal extension of vertical segment dilation led to concealed concretions or granulation tissue, posing challenges for detection via dacryoendoscopy. Among the 11 cases of canaliculitis complicated by dacryocystitis in this series, dacryoendoscopy failed to identify the concurrent presence of canaliculitis and dacryocystitis in 9 cases. The PPV and NPV for dacryoendoscopy were 100% and 89.93%, respectively.

CT-DCG showed very good consistency with the gold standard (kappa = 0.93), with a sensitivity of 94.4% and specificity of 98.5%. The 7 missed cases by CT-DCG were all attributed to small intracanalicular concretions that lacked typical imaging features such as filling defects or canalicular dilation. Only 2 non-canaliculitis cases (presenting with moderate canalicular dilation secondary to adjacent chalazions) were mistakenly diagnosed as canaliculitis, highlighting its superiority in minimizing diagnostic errors. Notably, CT-DCG correctly diagnosed all 11 cases of canaliculitis complicated by dacryocystitis. The PPV and NPV for CT-DCG were 98.35% and 94.96%, respectively.

## Discussion

### The challenge in diagnosing canaliculitis

Despite decades of literature detailing the constellation of clinical signs and symptoms, primary lacrimal canaliculitis remains frequently undiagnosed, misdiagnosed, and improperly treated even at present^17–19^. Canaliculitis has been reported to have a high misdiagnosis rate, ranging from 45% to 100% in previous literature^5^.

For those with atypical or ambiguous clinical symptoms, both diagnosis and differential diagnosis remains considerably challenging. In this study, both the canaliculitis group and the non-canaliculitis group had a high misdiagnosis rate when only routine slit-lamp examination was performed, which would subsequently result in a longer time required for accurate diagnosis. Inaccurate diagnosis may lead to inappropriate treatment and even potential adverse consequences. For instance, in a case report, a patient with canaliculitis was misdiagnosed as having conjunctivitis, and corneal perforation occurred due to the spread of infection^20^. In contrast, iatrogenic injury resulting from the misdiagnosis of non-canaliculitis can damage the tear pump function, leading to persistent epiphora^21^.

Some researchers have advocated microbiologic studies for diagnosing canaliculitis; however, the successful culture rate remains highly variable, ranging from 11.1% to 80.0%^5,6^. In this study, microbiological culture results were positive in 42.1% of cases. Although histopathologic analysis is highly sensitive for identifying pathogens, it may be misleading due to the morphologic similarities on staining between Actinomyces, Fusobacterium, Nocardiosis, Chromomycosis, and Botryomycosis^22^.In our study, Gomori methenamine silver staining and Giemsa staining identified specific Actinomyces species in 20 canaliculi from 16 eyes. Scholars have noted that the presence of concretions is diagnostic for canaliculitis^19,23^.

UBM is primarily used for high-resolution imaging of anterior segment structures, and there have been a few recent reports on its application in the diagnosis of canaliculitis^8,24^. UBM has limited imaging depth and a narrow field of view, which restricts its visualization to only the horizontal segment of the canaliculus, while failing to evaluate the lacrimal punctum, lacrimal sac, and nasolacrimal duct. This limits its clinical significance as a comprehensive diagnostic tool for canaliculitis^9^.

X-ray dacryocystography and radionuclide dacryoscintigraphy have insufficient spatial resolution to distinguish subtle structural abnormalities such as canalicular dilation, concretions, or mucosal irregularities. Consequently, they cannot reliably diagnose or differentially diagnose canaliculitis.

Dacryoendoscopy enables direct visualization of the canalicular lumen and offers higher diagnostic accuracy, making it a more clinically meaningful benchmark for evaluating the diagnostic value of CT-DCG. Therefore, this study selected dacryoendoscopy as the comparative tool. However, in our series, dacryoendoscopy failed to diagnose 15 cases of canaliculitis.This underperformance is primarily attributed to the anatomical challenges in visualizing the vertical segment of the canaliculus: concretions and granulation tissues in this region, due to their proximity to the lacrimal punctum, create a relative blind spot for dacryoendoscopy, impeding accurate detection. Additionally, in 11 cases of canaliculitis combined with dacryocystitis, it failed to identify concurrent involvement in 9 cases. Similarly, among 11 cases of combined superior-inferior canaliculitis, assessment of the contralateral canaliculus was neglected in 8 cases due to atypical symptoms, leading to incomplete diagnosis. Such limitations stem from its invasiveness, operator dependence, restricted field of view, and the risk of dislodging concretions—all of which hinder comprehensive evaluation of the LDS^25^.

### Dacryocystography and imaging characteristics of CT-DCG

In the early stage, imaging techniques such as X-ray and radionuclide dacyroscintigraphy were employed to assist in the diagnosis of canaliculitis^17,26–28^. In 2022, Yeh^15^ first used CT-DCG to examine the imaging features of 4 cases with secondary canaliculitis, revealing a characteristic “central radiolucency with marked surrounding soft-tissue enhancement”. To date, there have been no systematic studies using CT-DCG to observe the imaging features of primary canaliculitis.

In this study, the main imaging characteristics of CT-DCG include filling defects, dilation, raggedness, beading, and diverticula formation. In the canaliculitis group, dilation was observed in 127 out of 137 canaliculi; most dilation and concretions were distributed in the vertical segment of the canaliculus, followed by the proximal two-thirds of the horizontal segment. Dilation of the vertical segment may extend to varying degrees toward the temporal side, resulting in concealed concretions or granulation tissues—this poses challenges for detection via dacryoendoscopy and surgical intervention. CT-DCG can effectively display the overall morphological characteristics of the canaliculus and its dilation location, which aids in diagnostic suggestion.

Filling defect is another important imaging feature of canaliculitis. Among canaliculitis cases, 110 out of 137 canaliculi exhibited filling defects. We identified two distinct forms of filling defects: one adjacent to the canalicular wall, and the other in the central lumen—resulting in “central radiolucency with marked surrounding enhancement”, consistent with Yeh’s description^15^. These two types may sometimes coexist. Previous imaging studies^12^ have noted that filling defects, often indicating concretions, are a distinctive diagnostic feature of canaliculitis; our findings support this conclusion. This was confirmed by subsequent dacryoendoscopic examination, which showed that 110 affected canaliculi with filling defects corresponded to 108 concretions and 2 granulation tissues.

On CT-DCG images, “beading” is characterized by alternating narrowings and dilations along the canalicular lumen, resembling a string of beads. This pattern is defined as having at least 3 consecutive focal dilations separated by narrowed segments. During endoscopic examination, canaliculi with beading features were observed to have multiple segmental lesions.

Regarding small canalicular diverticula: CT-DCG analysis identified evident diverticula in the vertical segment of 13 canaliculi, which was confirmed by dacryoendoscopy. However, no apparent diverticula were observed in the horizontal segment across all cases. Literature reports mostly describe diverticula in the horizontal segment, which may be an illusion caused by the contrast between concretions/granulation-induced filling defects and surrounding contrast medium.

These features collectively provide a comprehensive morphological profile of primary canaliculitis, extending previous findings by focusing on primary rather than secondary cases.

On CT-DCG imaging, “raggedness” is characterized by irregular, serrated margins of the canalicular lumen, reflecting loss of the normal smooth contour. The raggedness of the canalicular wall serves as a secondary imaging feature that aids in the diagnosis of canaliculitis. This finding likely stems from chronic inflammatory insults, which induce mucosal edema with focal detachment and discontinuous epithelial regeneration—thereby producing irregular luminal contours on imaging.

In this group, 7 canaliculi showed atypical CT-DCG findings, characterized by slight dilation of the canalicular lumen.

In terms of patency: concretions may impede tear drainage, leading to epiphora. However, primary canaliculitis typically shows patency on syringing—this is also a common cause of misdiagnosis and delayed diagnosis. Patency on syringing can effectively exclude canaliculitis complicated by canalicular obstruction or dacryocystitis. Conversely, the presence of LDS obstruction does not definitively rule out concurrent canaliculitis, which poses a challenge to clinical diagnosis.

### Diagnostic value of CT-DCG

CT-DCG exhibited exceptional diagnostic performance, with very good consistency with the gold standard (kappa = 0.93), high sensitivity (94.4%), and specificity (98.5%). Its superiority is manifested in several critical aspects:

CT-DCG missed 7 cases, all involving small intracanalicular concretions lacking typical features (e.g., filling defects or dilation), and incorrectly diagnosed 2 non-canaliculitis cases (secondary to chalazion-induced dilation). This low rate of false negatives and positives directly addresses the high misdiagnosis rate observed in conventional methods, reducing the risk of inappropriate treatment.

Approximately 2.9%-8.3% of patients with canaliculitis present with concurrent dacryocystitis^29,30^. Additionally, canaliculitis may sometimes involve both the superior and inferior canaliculi simultaneously. These circumstances necessitate a comprehensive evaluation of both the canaliculi and the lacrimal sac to achieve an accurate diagnosis. Unlike slit-lamp examination and dacryoendoscopy, CT-DCG effectively identifies combined lesions. It accurately identified all 11 cases of canaliculitis complicated by dacryocystitis (defined by canalicular dilation, filling defects, nasolacrimal duct obstruction, and lacrimal sac dilation) and all 11 cases of combined superior-inferior canaliculitis. This contrasts sharply with slit-lamp examination (detecting 2/11 combined superior-inferior cases) and dacryoendoscopy (fully evaluating 3/11 combined superior-inferior cases and 2/11 combined dacryocystitis cases). By visualizing the entire LDS morphology (e.g., dilation, filling defects, beading, and diverticula), CT-DCG overcomes the anatomical blind spots of endoscopy and the superficial observation of slit-lamp examination, thereby guiding more precise management. Specifically, in cases of concurrent superior and inferior canaliculitis, lacrimal canaliculotomy is performed simultaneously. For cases with both canaliculitis and dacryocystitis, clinicians appropriately abandoned simple lacrimal canaliculotomy and opted instead for a combined surgical approach: lacrimal canaliculotomy combined with dacryocystorhinostomy. This strategy effectively avoided secondary surgeries, improved treatment success rates, shortened patients’ recovery periods, and reduced medical costs.

The prolonged median time to diagnosis (10 months) in this cohort highlights the need for a reliable diagnostic tool. CT-DCG’s ability to provide detailed, non-invasive imaging of the canaliculi enables early identification of pathognomonic features, shortening the diagnostic window and reducing disease progression.

In routine clinical practice, the primary challenge in diagnosing primary lacrimal canaliculitis lies in distinguishing it from other diseases with overlapping clinical manifestations, such as conjunctivitis, dacryocystitis, or blepharitis. These conditions share common symptoms including epiphora, medial canthal erythema, and punctal discharge, which are precisely the root causes of diagnostic uncertainty and misdiagnosis. Therefore, by selecting the non-canaliculitis group as the control, we aimed to test the ability of CT-DCG to identify canaliculitis in the context of complex pathological conditions, thereby reflecting its performance in real-world scenarios where clinicians have to deal with ambiguous symptoms. This approach directly addresses the core clinical need: verifying whether CT-DCG can reduce misdiagnosis by differentiating canaliculitis from its most similar conditions. Notably, patients were enrolled in the non-canaliculitis group only after completing a comprehensive preliminary evaluation, which included slit-lamp examination and lacrimal irrigation. If the preliminary assessment clearly ruled out canaliculitis (e.g., no epiphora, no mucoid discharge, and no medial canthal erythema or nodules), CT-DCG and subsequent related examinations were not performed. This targeted approach ensured we avoided unnecessary imaging while maintaining the rigor and validity of the control group.

Despite the promising findings, this study has several limitations. First, the retrospective design across three centers, while enhancing generalizability to some extent, may still introduce selection bias, as consecutive cases may not fully represent the broader spectrum of primary canaliculitis. Second, while larger than previous studies, the sample size remains modest, requiring validation in larger multiethnic cohorts to confirm CT-DCG’s performance in subtle or atypical lesions. Third, secondary canaliculitis cases (e.g., punctal plug-related) were excluded, limiting understanding of how CT-DCG distinguishes primary from secondary forms. Fourth, the impact of cutaneous fistulae (present in 4 cases) on pathogenesis remains unclear. Fifth, the study lacks in-depth analysis of the correlation between specific CT-DCG features (e.g., the size or location of filling defects) and long-term clinical outcomes such as recurrence rates or surgical complication risks, which could strengthen its prognostic value. Sixth, the study did not incorporate dynamic CT-DCG monitoring post-treatment or analyze the link between evolving imaging features and treatment response. Additionally, the cost-effectiveness of CT-DCG compared to more affordable modalities (e.g., basic UBM) was not evaluated, which may influence its adoption in resource-constrained environments.

Looking forward, targeted efforts to address these limitations could extend the clinical impact of our work:

To address selection bias from the retrospective design, future studies should adopt a prospective, multi-center framework to enroll a more representative cohort, ensuring findings generalize to diverse clinical settings.

To overcome the modest sample size, large-scale multiethnic studies are needed to validate CT-DCG’s sensitivity for subtle lesions (e.g., small concretions) and refine diagnostic thresholds for atypical cases.

To fill the gap in distinguishing primary vs. secondary forms, upcoming research should include secondary canaliculitis cohorts, comparing their CT-DCG features with primary cases to identify discriminatory imaging signatures.

To clarify the role of cutaneous fistulae, mechanistic studies (e.g., microbiological analysis of fistula secretions) should explore whether fistulae contribute to disease persistence or recurrence, informing targeted interventions.

To address the lack of post-treatment dynamic analysis, future studies should implement serial CT-DCG follow-up in treated patients.

To enhance clinical utility, future work should: analyze correlations between CT-DCG features (e.g., beading, diverticula) and long-term outcomes (recurrence, surgical complications) to establish prognostic value; conduct cost-effectiveness analyses against UBM to guide resource allocation in low-resource settings.

Another crucial task is developing a standardized diagnostic algorithm based on key CT-DCG features (e.g., filling defects, dilation patterns). This would streamline interpretation, reduce variability, and facilitate routine clinical use. Together, these efforts aim to fully translate the diagnostic potential of CT-DCG into improved clinical outcomes for patients with canaliculitis.

## Conclusions

The high misdiagnosis rate and prolonged diagnostic delay in canaliculitis underscore the limitations of current methods. CT-DCG, with its superior sensitivity, specificity, and capacity to evaluate complex and atypical cases, addresses these shortcomings. Its ability to minimize diagnostic errors, offer clear visualization of the unique imaging characteristics of canaliculitis, visualize the entire LDS, and guide appropriate treatment (e.g., combined surgery for concurrent dacryocystitis or combined superior-inferior canaliculitis) establishes it as a highly valuable diagnostic tool. CT-DCG offers a novel and effective approach, with significant promise in improving diagnostic accuracy, reducing patient recurrence rates, and optimizing the clinical management of canaliculitis. Overall, CT-DCG shows great promise in enhancing the diagnosis of primary lacrimal canaliculitis and guiding clinical treatment.

## Data Availability

The data that support the findings of this study are available from the corresponding author (Lihong Yang, ylhong922@126.com) upon reasonable request.
